# Editorial: Pulmonary involvement in systemic autoimmune rheumatic diseases (SARDs): from diagnostic tools to therapeutic strategies

**DOI:** 10.3389/fmed.2025.1720761

**Published:** 2025-10-30

**Authors:** Eirini Vasarmidi, Maria Otaola, Pierre-Antoine Juge

**Affiliations:** ^1^Department of Respiratory Medicine and Laboratory of Molecular and Cellular Pulmonology, School of Medicine, University of Crete, Heraklion, Greece; ^2^Rheumatology Section, Instituto de Rehabilitación Psicofísica (IREP), Buenos Aires, Argentina; ^3^Hopital Bichat - Claude-Bernard Service de Rhumatologie, Paris, France

**Keywords:** systemic autoimmune rheumatic diseases, connective tissue disease, interstitial lung disease, pulmonary fibrosis, rheumatoid arthritis, inflammatory myopathies, myositis, bronchoalveolar lavage

Pulmonary involvement is a major cause of morbidity and mortality among patients with Systemic Autoimmune Rheumatic Diseases (SARDs), including rheumatoid arthritis (RA), systemic sclerosis (SSc), systemic lupus erythematosus (SLE), and inflammatory myopathies (IIM) (1). The respiratory system can be affected in several ways—airway, pleural, or vascular—but the most clinically significant manifestation is Interstitial Lung Disease (ILD), termed SARD-ILD (2, 3). This condition is characterized by inflammation and fibrosis of the lung interstitium, leading to cough, progressive dyspnea, and impaired gas exchange. The clinical course is variable, with some cases evolving into Progressive Pulmonary Fibrosis (PPF), a severe and often fatal complication (4).

Management of pulmonary disease in SARDs is complex and requires a multidisciplinary approach involving rheumatologists, pulmonologists, and radiologists. Diagnosis combines Pulmonary Function Tests (PFTs)—notably forced vital capacity (FVC) and diffusing capacity for carbon monoxide (DLCO)—with High-Resolution Computed Tomography (HRCT), which provides critical diagnostic and prognostic information about ILD patterns (4, 5).

This Research Topic aimed to highlight advances in detecting lung involvement in SARDs and to promote personalized management strategies. Two studies focused on CT-based lung evaluation.

Yang et al. conducted a retrospective study of 591 patients with a usual interstitial pneumonia (UIP) pattern, including 229 with SARD-UIP. They compared outcomes across different etiologies and found marked heterogeneity. Patients with idiopathic pulmonary fibrosis (IPF) experienced a more rapid FVC decline than those with SARD-UIP, while within connective tissue diseases, primary Sjögren's syndrome (pSS)-UIP progressed more slowly than RA-UIP and vasculitis-UIP. Survival among RA-UIP and ANCA-associated vasculitis (AAV)-UIP was similar to IPF. The authors suggested that UIP etiology should be considered in trial design, although larger studies are needed, as some data indicate similar clinical behavior regardless of cause (6).

Qin et al. advanced SARD-ILD prognosis using CT radiomics to estimate disease severity. The GAP index (gender, age, pulmonary physiology) is a known mortality predictor in ILD (7). Their radiomics model accurately distinguished ILD-GAP stages (AUC > 0.8 across all cohorts). Unlike subjective HRCT interpretation, radiomics provides objective, quantitative assessment. Their radiomics nomogram outperformed visual evaluation in staging SARD-ILD, suggesting this noninvasive method could meaningfully enhance clinical decision-making.

Among SARDs, rheumatoid arthritis (RA) is most prevalent, affecting about 1% of the global population. RA-associated ILD (RA-ILD) is a major mortality driver—second only to cardiovascular disease (8, 9)—with a 5-year mortality near 40%.

A review by Bernardinello et al. summarized genetic risk factors in RA-ILD. The MUC5B promoter variant (rs35705950) emerged as a key risk allele, especially in patients with a UIP pattern (10). Rare variants in telomerase genes (TERT, RTEL1, PARN) are enriched in RA-ILD, and shorter telomeres correlate with more severe disease. Specific HLA alleles also influence risk: DRB1*16 and DQB1*06 increase susceptibility, while DRB1*04 and DQB1*04 appear protective. To validate these associations and explore therapeutic implications, large multiethnic cohorts and genotype-stratified clinical trials (e.g., by MUC5B or telomerase status) are needed to assess responses to antifibrotics and biologics.

On the other hand, Neofotistou-Themeli et al. reviewed fibroblast biology, noting that single-cell sequencing has revealed diverse fibroblast subpopulations within RA synovium and lung tissue. These cells exhibit distinct phenotypes that drive inflammation, extracellular matrix remodeling, and fibrosis, contributing to relapses and treatment resistance. Targeting fibroblast subsets—such as through Notch signaling modulation or migration inhibition—may complement current immunotherapies. Together, these studies depict RA-ILD as the product of systemic autoimmunity, fibroblast-driven fibrosis, and genetic predisposition. Integrating molecular and genetic insights could enable earlier diagnosis and targeted, genotype-informed interventions to improve outcomes.

Among the most challenging forms of SARD-ILD are those linked to inflammatory myopathies (IIM) (11, 12). Myositis-associated ILD can precede or occur without muscle involvement in up to 40% of patients (13–15), complicating diagnosis.

Tzilas et al. retrospectively analyzed 35 patients with clinically amyopathic ILD and myositis-specific autoantibodies (MSAs). None had muscle weakness, and only 11% showed elevated creatine kinase. The predominant HRCT patterns were nonspecific interstitial pneumonia (NSIP) (49%) and NSIP/organizing pneumonia (OP) overlap (39%). Cutaneous signs such as “mechanic's hands” and Gottron papules were infrequent.

Autoantibody analysis showed anti–aminoacyl-tRNA synthetase (anti–tRNA-syn) antibodies in 80% of patients, most commonly anti–Jo-1 (54%). The authors concluded that amyopathic ILD with MSAs should be suspected in patients presenting with NSIP or NSIP/OP patterns, even without clinical myositis. Bronchoalveolar lavage fluid (BALF) revealed lymphocytosis ≥20% in 65% (median 24%) and a low CD4/CD8 ratio (mean 0.38).

Conversely, Zhang et al. examined the microbial diversity in BALF from IIM-ILD patients using metagenomic next-generation sequencing (mNGS). They compared 20 IIM-ILD cases with 16 non-IIM SARD-ILD and 15 community-acquired pneumonia (CAP) patients. Untreated IIM-ILD patients exhibited lower BALF lymphocytes (9.15 ± 17.18) and higher neutrophils than in Tzilas et al.'s study, along with mild leukocytosis and elevated CRP, suggesting potential infectious contributions. Microbiome profiling revealed distinct differences: the IIM-ILD group showed higher abundance of *Pseudomonas* and *Corynebacterium*—especially *Pseudomonas aeruginosa*—while *Prevotella pallens* predominated in non-IIM CTD-ILD.

Although both studies were limited by small cohorts, they emphasize the need for larger investigations to clarify the alveolar immune and microbial environment in IIM-ILD and its prognostic implications.

Yao et al. performed a meta-analysis of 50 cohort studies to identify determinants of ILD progression across SARDs. They found that disease worsening was associated with male sex, a UIP pattern, extensive lung involvement, and advanced age, while rapid progression correlated with elevated C-reactive protein (CRP), Ro52, and MDA5 antibody positivity. These findings underscore the importance of integrating demographic, radiological, and serological factors into risk stratification.

**Figure 1:**
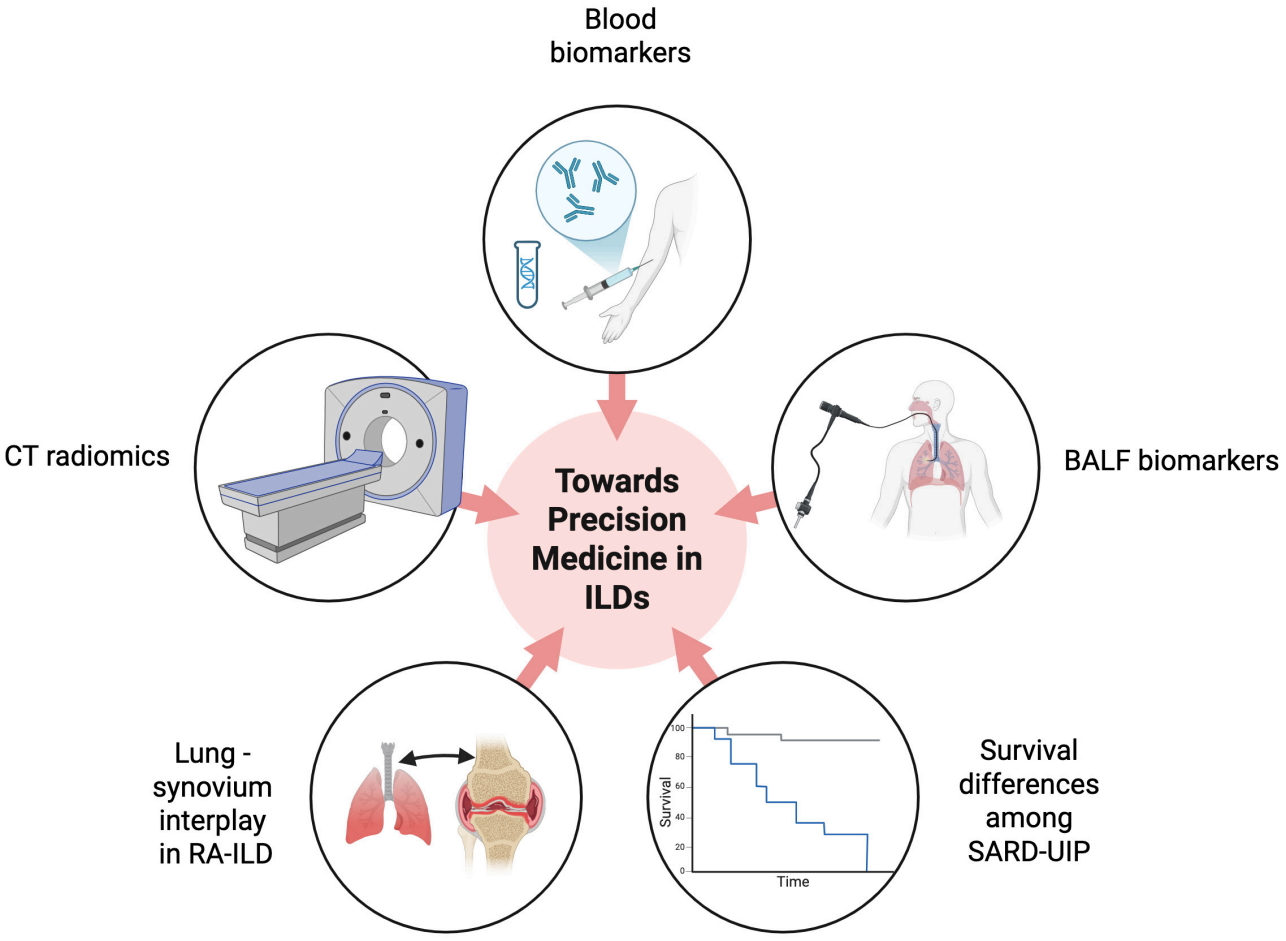
Integrating demographics, radiologic findings, blood biomarkers including genetic data, as well as BALF lymphocytosis and alveolar microbiome, will enhance the diagnosis, evaluation and prognosis assessment of patients with SARD-ILD, moving toward precision medicine. Created in https://BioRender.com. CT, computed tomography; BALF, bronchoalveolar lavage fluid; SARD, systemic-autoimmune rheumatic diseases; ILD, interstitial lung disease; RA, rheumatoid arthritis.

In summary, this Research Topic reflects a paradigm shift in SARD-ILD evaluation: from descriptive diagnosis toward quantitative, individualized assessment. Noninvasive or minimally invasive tools—such as CT radiomics, BALF profiling, and emerging molecular biomarkers—are central to this evolution. Combining these with established functional and imaging data within a multidisciplinary framework will accelerate the transition toward precision medicine in ILD (Figure 1). Ultimately, integrating genetic, cellular, and radiologic markers will allow earlier detection, more accurate prognostication, and tailored therapies, improving both survival and quality of life for patients with SARD-ILD.
